# MITEA: A dataset for machine learning segmentation of the left ventricle in 3D echocardiography using subject-specific labels from cardiac magnetic resonance imaging

**DOI:** 10.3389/fcvm.2022.1016703

**Published:** 2023-01-10

**Authors:** Debbie Zhao, Edward Ferdian, Gonzalo D. Maso Talou, Gina M. Quill, Kathleen Gilbert, Vicky Y. Wang, Thiranja P. Babarenda Gamage, João Pedrosa, Jan D’hooge, Timothy M. Sutton, Boris S. Lowe, Malcolm E. Legget, Peter N. Ruygrok, Robert N. Doughty, Oscar Camara, Alistair A. Young, Martyn P. Nash

**Affiliations:** ^1^Auckland Bioengineering Institute, University of Auckland, Auckland, New Zealand; ^2^Department of Anatomy and Medical Imaging, University of Auckland, Auckland, New Zealand; ^3^Institute for Systems and Computer Engineering, Technology and Science (INESC TEC), Porto, Portugal; ^4^Department of Cardiovascular Sciences, KU Leuven, Leuven, Belgium; ^5^Counties Manukau Health Cardiology, Middlemore Hospital, Auckland, New Zealand; ^6^Green Lane Cardiovascular Service, Auckland City Hospital, Auckland, New Zealand; ^7^Department of Medicine, University of Auckland, Auckland, New Zealand; ^8^Department of Information and Communication Technologies, Universitat Pompeu Fabra, Barcelona, Spain; ^9^Department of Biomedical Engineering, King’s College London, London, United Kingdom; ^10^Department of Engineering Science, University of Auckland, Auckland, New Zealand

**Keywords:** 3D echocardiography (3DE), machine learning (ML), segmentation (image processing), left ventricle (LV), multimodal imaging, cardiac magnetic resonance (CMR) imaging, domain adaptation, Cardiac Atlas Project

## Abstract

Segmentation of the left ventricle (LV) in echocardiography is an important task for the quantification of volume and mass in heart disease. Continuing advances in echocardiography have extended imaging capabilities into the 3D domain, subsequently overcoming the geometric assumptions associated with conventional 2D acquisitions. Nevertheless, the analysis of 3D echocardiography (3DE) poses several challenges associated with limited spatial resolution, poor contrast-to-noise ratio, complex noise characteristics, and image anisotropy. To develop automated methods for 3DE analysis, a sufficiently large, labeled dataset is typically required. However, ground truth segmentations have historically been difficult to obtain due to the high inter-observer variability associated with manual analysis. We address this lack of expert consensus by registering labels derived from higher-resolution subject-specific cardiac magnetic resonance (CMR) images, producing 536 annotated 3DE images from 143 human subjects (10 of which were excluded). This heterogeneous population consists of healthy controls and patients with cardiac disease, across a range of demographics. To demonstrate the utility of such a dataset, a state-of-the-art, self-configuring deep learning network for semantic segmentation was employed for automated 3DE analysis. Using the proposed dataset for training, the network produced measurement biases of −9 ± 16 ml, −1 ± 10 ml, −2 ± 5 %, and 5 ± 23 g, for end-diastolic volume, end-systolic volume, ejection fraction, and mass, respectively, outperforming an expert human observer in terms of accuracy as well as scan-rescan reproducibility. As part of the Cardiac Atlas Project, we present here a large, publicly available 3DE dataset with ground truth labels that leverage the higher resolution and contrast of CMR, to provide a new benchmark for automated 3DE analysis. Such an approach not only reduces the effect of observer-specific bias present in manual 3DE annotations, but also enables the development of analysis techniques which exhibit better agreement with CMR compared to conventional methods. This represents an important step for enabling more efficient and accurate diagnostic and prognostic information to be obtained from echocardiography.

## 1. Introduction

Machine learning (ML) has shown considerable promise for automated analysis and interpretation in the domain of cardiovascular imaging (1, 2). Already, its application to cardiac magnetic resonance (CMR) imaging has exhibited excellent results with high accuracy and reproducibility by leveraging several large cohort databases such as the UK Biobank (3–5). Although CMR offers higher spatial resolution and tissue contrast for the assessment of cardiac mass and volume, transthoracic echocardiography remains at the frontline of cardiac imaging as the most widely used and readily accessible modality for screening, diagnosis, and management of cardiovascular disease. Technological advances in ultrasonography have enabled three-dimensional echocardiography (3DE), consequently removing the dependency on accurate plane positioning and geometric assumptions required for standard two-dimensional echocardiography (2DE). As a result, several studies have shown that 3DE-derived measurements are generally superior to 2DE in terms of chamber quantification accuracy (6, 7), reproducibility (8), and prognostic power (9). Despite these advantages, 3DE has not yet been universally integrated into clinical practice for the assessment of cardiac function due to limitations in image quality, and increased costs associated with acquisition and long analysis times compared with 2DE.

In comparison to other cardiac imaging modalities, analysis of 3DE is particularly challenging owing to the limited spatial resolution, low contrast-to-noise ratio (CNR), complex noise characteristics (speckle in combination with common artifacts), and image anisotropy. Several factors can influence the image quality of 3DE including, but not limited to, sonographer experience, vendor-specific processing, acquisition settings, and patient body habitus. Discrepancies in the delineation of important cardiac structures, such as the left ventricle (LV), compared to those from a reference modality such as CMR, have been shown to be observer- and software-dependent, as well as exhibit regional variability in terms of the magnitude of differences in geometry (10). In particular, acoustic shadowing and signal dropout further compromise local image quality, leading to greater inter- and intra-observer variability in manual annotations at these locations. To address this, statistical shape priors (or atlases) can be used to provide suitable estimates in regions where image information is corrupted or missing (11–14). However, these approaches are ultimately limited by the generalizability of such templates and may be ill-suited in cases of atypical anatomy.

The primary challenge associated with the development of automated methods for 3DE analysis is the prerequisite of a sufficiently large training dataset. Historically, reference annotations have been difficult to obtain due to the high degree of variability associated with manual 3DE segmentation, thus, limiting the scope of ML-based solutions. Currently, the dataset belonging to the Challenge on Endocardial Three-dimensional Ultrasound Segmentation (CETUS)^1^ (15), organized as part of the 2014 Medical Image Computing and Computer Assisted Interventions (MICCAI) conference, remains the only publicly available resource. This dataset consists of expert-annotated 3DE images from 45 subjects, for which data from 15 subjects are made publicly available for training. Due to the lack of clear guidelines for endocardial contouring in 3DE, considerable effort was expended in establishing a consistent analysis regime amongst three expert observers. Despite this, large inter-expert variability was reported and an agreement was only reached after several revisions and consensus discussions (16). Nevertheless, efforts in providing a publicly accessible benchmark such as the CETUS platform represent an important step toward the development of automated 3DE analysis methods.

Alternative approaches for generating training data involve producing synthetic 3DE images via *in silico* simulations (17, 18) or generative adversarial networks (19, 20). While these methods do not require additional segmentation (as the underlying anatomy is known in such cases), synthetic datasets are often unable to adequately capture all features found in real images. Unsupervised domain adaptation strategies have also gained interest in medical imaging applications, enabling knowledge gained from higher-resolution images or data to improve the segmentation of lower-resolution or degraded images (21–23). However, as with unsupervised methods in general, it cannot be certain that the model is optimized for the target domain.

Alongside the ongoing advances in 3DE acquisition systems, more accurate and efficient analysis methods will substantially benefit patient care and management. Having acknowledged the lack of expert consensus in obtaining reference annotations, and the limitations associated with population shape priors and synthetic data, we instead leveraged subject-specific labels from CMR acquired in a heterogenous population of 134 subjects. Here, we present MITEA (MR-Informed Three-dimensional Echocardiography Analysis): an annotated 3DE dataset for the segmentation of the LV myocardium and cavity for quantification of systolic function and mass, and subsequently show how this data can be used to train a deep learning model for automated 3DE analysis. The full annotated 3DE dataset and trained model can be accessed as part of the Cardiac Atlas Project^2^ (24). To date, this represents the largest publicly available 3DE dataset, and the first which uses labels derived from subject-specific CMR analyses.

## 2. Materials and methods

Non-invasive multimodal 3DE and CMR imaging were performed within two hours in 144 prospectively recruited participants (87 healthy subjects with no existing or history of cardiac disease; and 57 patients with acquired, non-ischemic cardiac disease), of which 134 (82 healthy subjects; and 52 patients with cardiac disease) were included in the study. Ethical approval for this research was granted by the Health and Disability Ethics Committee of New Zealand (17/CEN/226). Written informed consent was obtained from each participant.

**FIGURE 1:**
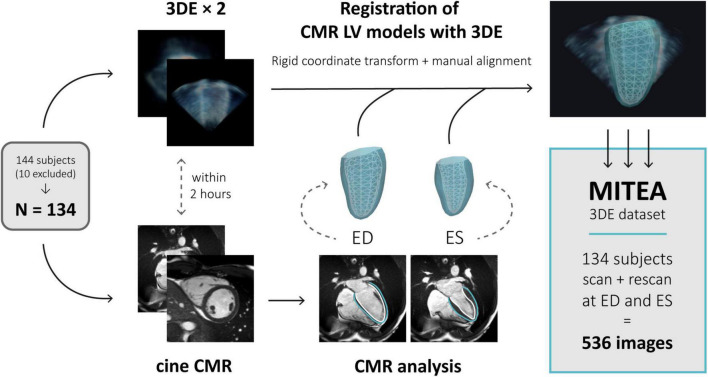
Method overview for generation of the MR-Informed Three-dimensional Echocardiography Analysis (MITEA) dataset, showing paired multimodal imaging using 3D echocardiography (3DE) and cardiac magnetic resonance (CMR) imaging. The registration of CMR-derived left ventricular geometries was performed at end-diastole (ED) and end-systole (ES) to produce subject-specific labels for the myocardium and cavity.

Multimodal data belonging to 70 of these subjects have been previously presented as part of an investigation into systematic measurement biases between 3DE and CMR (10). The present study extends upon this work by: inclusion of additional disease cases for improved generalizability; inclusion of scan-rescan 3DE images to assess repeatability; and utilization of paired multimodal data for the development of automated 3DE segmentation techniques. An overview of the method for data generation is illustrated in Figure 1, and detailed in the following subsections.

### 2.1. Multimodal image acquisition

Transthoracic real-time (single-cycle) 3DE images were acquired using a Siemens ACUSON SC2000 Ultrasound System and a 4Z1c matrix array transducer (Siemens Medical Solutions, Mountain View, CA, USA) with 36 × 48 (1,728) elements. Targeted images of the LV were acquired from the apical window in a steep left lateral decubitus position during breath-holds. Parameters (including choice of fundamental or harmonic imaging, depth, gain, compression, and width of the volumetric dataset) were optimized by an experienced sonographer on a per-subject basis to maximize the image volume sampling rate, while maintaining adequate spatial resolution for analysis. To measure scan-rescan repeatability, two 3DE clips were acquired per subject (producing a total of 268 3DE datasets across the 134 included participants). All acquisitions were reconstructed into 3D Cartesian image volumes (with a rectangular bounding box and zero-values outside the pyramidal volume) using 1 mm isotropic voxels.

Multi-planar cine CMR imaging was performed on either a Siemens Magnetom 1.5T Avanto Fit (*n* = 77) or 3T Skyra (*n* = 57) scanner (Siemens Healthcare, Erlangen, Germany) with an 18-channel body matrix coil, using a retrospectively gated balanced steady-state free precession sequence under breath-holds. Acquired planes included three long-axis slices (standard two-, three-, and four-chamber views) and a short-axis stack of 6–10 slices (spanning the length of the LV from mitral valve to apex) over one cardiac cycle, with the following typical imaging parameters: TR = 3.7 ms, TE = 1.6 ms, flip angle = 45°, field of view = 360 mm × 360 mm, in-plane resolution = 1.4 mm × 1.4 mm, and slice thickness = 6 mm, in keeping with standard protocols. With these settings, an average of 29 ± 4 (range 20–44) frames per cardiac cycle were obtained for the included study population.

### 2.2. Image analysis

Patients were subjectively graded on a five-point 3DE image quality scale (poor, suboptimal, adequate, good, excellent) by a single expert (independent of the sonographer who acquired the images). This subjective score was based on a combination of perceived endocardial border definition (i.e., the overall sharpness of the LV cavity due to ultrasound attenuation, choice of harmonics, and selection of gains and compression), and the visibility of wall segments (relating to signal dropout and LV coverage due to probe alignment and selection of an adequate pyramidal volume size). After qualitative grading, the ratio between the mean difference and variance in signal intensity between the LV myocardium and cavity were calculated to provide a quantitative measure of CNR (25), given by:

CNR=|μmyocardium-μcavity|σmyocardium2+σcavity2

where μ_myocardium_ and μ_cavity_ are the mean signal intensities in the regions belonging to the myocardium and cavity, respectively, and σ_myocardium_ and σ_cavity_ are the corresponding standard deviations.

**FIGURE 2:**
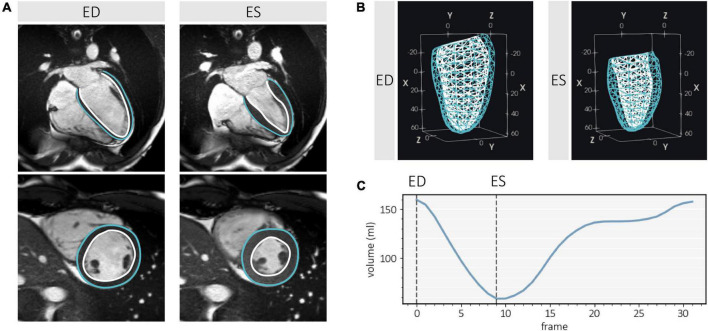
Image analysis and 3D left ventricle (LV) geometry extraction from cardiac magnetic resonance (CMR) using Cardiac Image Modeler (CIM, Version 8.1, University of Auckland, New Zealand) at end-diastole (ED) and end-systole (ES). **(A)** Contour examples of the endocardium (white) and epicardium (blue) on a 4-chamber long axis slice and mid-ventricular short axis slice, showing exclusion of trabeculae and papillary muscles from the myocardium. **(B)** 3D surface meshes (dimensions in mm) sampled from the LV finite element model. **(C)** Volume-time curve generated from CMR image analysis, indicating frame indices of interest.

To generate subject-specific labels from CMR, time-varying geometric models of the LV over one cardiac cycle were constructed semi-automatically by guide-point modeling (26) using *Cardiac Image Modeler* (CIM, Version 8.1, University of Auckland, New Zealand), by a single analyst. To create an initial coarse geometry and to establish the LV position and orientation, fiducial landmarks (i.e., the base of the myocardium in the long-axis slices; apical and basal centroids in the corresponding short axis slices, and insertion points of the right ventricle (RV) along the LV epicardial border in the short-axis slices, where visible) were manually identified. This was subsequently refined by interactively fitting contours to the endocardial and epicardial borders on both the long- and short-axis slices, and manually correcting in-plane breath-hold mis-registrations using the image intersections. Papillary muscles and trabeculations were included within the LV cavity (Figure 2A). This analysis generated a bicubic Hermite and linear finite element model of the LV (27), with the origin positioned at one-third of the distance from base to apex, with the LV long axis parallel to the *x*-axis, and the center of the RV directed toward the orthogonal *y*-axis. From the model, 145 unique points were sampled per surface (for the endocardium and epicardium) to produce a mesh consisting of 290 3D rectangular Cartesian (*x*, *y*, *z*) vertices representing the LV myocardium. Static 3D LV geometries were extracted at end-diastole (ED) and end-systole (ES) (Figure 2B), corresponding to the first CMR image frame, and the image frame associated with the smallest cavity volume, respectively (Figure 2C).

### 2.3. Multimodal registration and label generation

For each subject, registration of CMR with 3DE was performed in two steps, comprising an automated coarse alignment of the global LV position, followed by a manual refinement of the LV model within the 3DE image volume. To establish the initial transform at ED, the B-spline Explicit Active Surfaces (BEAS) algorithm (14) was used to create a fully automated segmentation of the LV from 3DE, from which a vector connecting the apex and basal centroid was extracted to represent the LV long axis orientation and position with respect to each 3DE acquisition. To differentiate between the circumferential wall segments, the direction of the RV center from the central axis was approximated as being 70 degrees from the inferior RV insertion [automatically detected based on image features as part of the BEAS segmentation (28)], as the anterior insertion is generally not well visualized in 3DE. The resultant axes were subsequently registered to the cardiac coordinate system used in the finite element model of the LV in Section “2.2 Image analysis,” yielding a transformation matrix representing the rigid mapping between the 3DE image LV model coordinate systems. This transformation was subsequently applied to initially align the CMR-derived LV model to the 3DE image for each subject.

**FIGURE 3:**
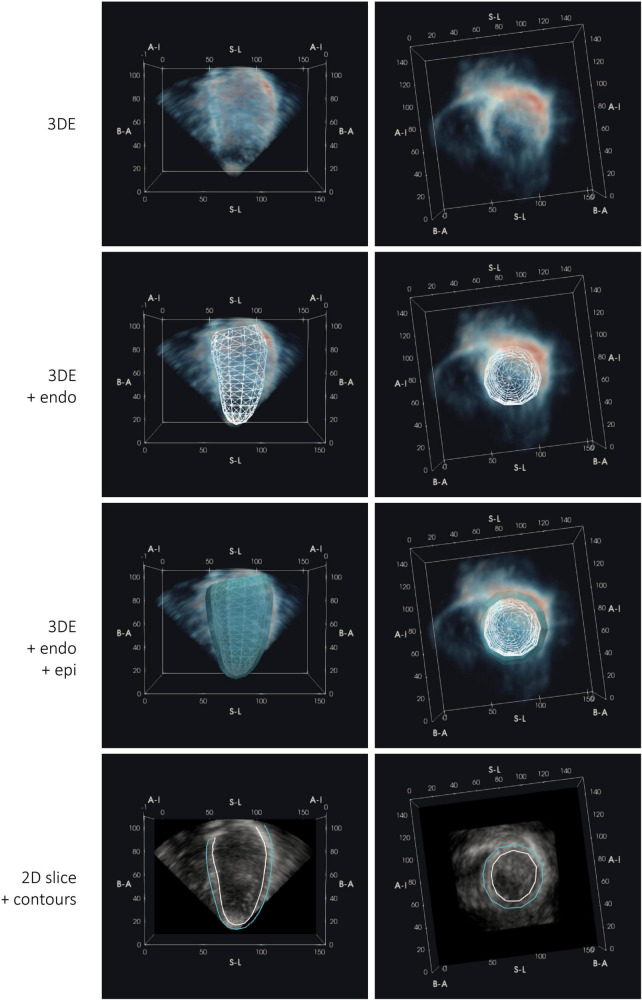
Registration of 3D echocardiography (3DE) with subject-specific geometries of the left ventricle (LV) derived from cardiac magnetic resonance (CMR) at end-diastole, showing an example 3DE image volume (visualized with an opacity transfer function on a blue-to-red colormap) and corresponding 2D mid-ventricular image slice and contours of the endocardium (endo) and epicardium (epi), viewed longitudinally and axially. Labels denote anatomical LV aspects: B-A, base-to-apex; S-L, septal-lateral; A-I, anterior-inferior. All dimensions are in mm.

The initial alignment was refined by manually applying rigid translations and rotations using an open-source data analysis and visualization application (ParaView 5.8.0) (29) (Figure 3), by the same expert that carried out subjective 3DE image quality grading and CMR analysis. Manual registrations were performed at two frames only, representing ED and ES. For CMR, the relevant static LV geometries were extracted according to the method described in Section “2.2 Image analysis” and Figure 2C. For 3DE, ED and ES image frames were manually selected corresponding to when the cavity appeared largest and smallest. The manual refinement was performed independently for the ED frame, and further adjusted at ES, as required, to account for any changes in relative transducer angle and position over the cardiac cycle during acquisition. All manual alignments were carried out by a single observer, resulting in 536 (134 included subjects × 2 clips × 2 frames) independent alignments.

**FIGURE 4:**
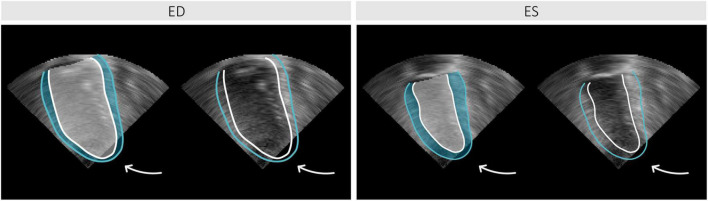
Example of an annotated 3D echocardiography (3DE) image sliced longitudinally at end-diastole (ED) and end-systole (ES), showing portions of labeled regions and corresponding contours for the left ventricular cavity (white) and myocardium (blue) falling outside the acquired 3DE pyramidal volume (as indicated by the arrows).

The closed meshes were subsequently converted into 3D masks of equal dimensions to the corresponding Cartesian 3DE images, containing two foreground label classes (representing the cavity and myocardium). Of note, foreground label regions were not constrained to the pyramidal volume, as shown in Figure 4.

### 2.4. Deep learning segmentation experiment

To demonstrate the application of the dataset for deep learning, *nnU-Net* (30), a self-configuring network for semantic segmentation was employed for automated 3DE analysis. An 80/20 split was used for training and testing, with images from the same acquisition (i.e., ED and ES from the same cycle) and clips from the same participant (i.e., scan and rescan) grouped together. This resulted in data from 107 unique participants being included in the training set (a total of 428 paired images and labels), and data from 27 participants in the testing set (108 paired images and labels). The network was trained using fivefold cross-validation with a further 80/20 split for training and validation, producing five model instances (each trained using data from 85 or 86 participants), which were ensembled (by averaging softmax probabilities) for inference.

**FIGURE 5:**
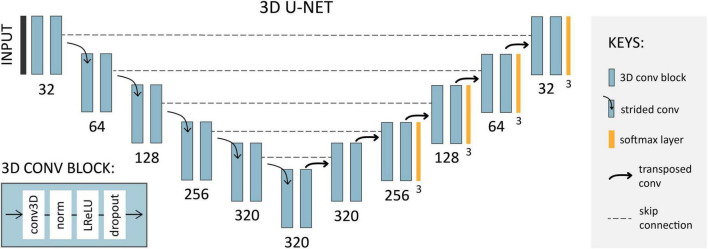
Network architecture configured by *nnU-Net* for 3DE segmentation. Each 3D convolution (conv) block consists of a plain convolution, followed by instance normalization (norm), leaky ReLU (LReLU), and dropout. Downsampling is achieved using strided convolutions (stride two), and upsampling by transposed convolutions. Numbers indicate the number of channels corresponding to each convolution block.

Using the 3D full-resolution U-Net configuration with no cascade, each fold was trained for 200 epochs (chosen empirically based on stable validation loss curves), where each epoch consisted of 250 iterations over shuffled batches of size two. Stochastic gradient descent with a large Nesterov momentum (31) (μ = 0.99) and a high initial learning rate of 0.01 [reduced by (1 − epoch_current_/epoch_max_)^0.9^] using the *polyLR* schedule (32), producing an almost-linear decrease to zero, was used for optimization, with the sum of cross-entropy and Dice as the loss function. To diversify the data and increase model robustness, on-the-fly data augmentations including rotation, scaling, mirroring, and low-resolution simulation (by means of downsampling followed by upsampling), were applied during training. Training time was approximately 170 s per epoch on an NVIDIA Tesla V100 GPU with 32GB memory. With the exception of a reduction in the number of epochs (set to 1,000 by default) to reduce overfitting, the model was deployed with all other out-of-the-box parameters for pre-processing, network architecture selection, training, and post-processing. The self-configured architecture for the present dataset is shown in Figure 5.

### 2.5. Validation and performance

Model performance was evaluated on the testing set (*n* = 27 subjects, 54 acquisitions) in terms of segmentation accuracy at ED and ES using the Dice coefficient, mean surface distance (MSD), and Hausdorff distance (HD); as well as the agreement in routine clinical cardiac indices including LV end-diastolic volume (EDV), end-systolic volume (ESV), mass (LVM) (calculated as the average of mass at ED and ES), and ejection fraction (EF). Clinical measurements were also compared with those derived from conventional manual analysis using TOMTEC 4D LV-ANALYSIS 3 (TOMTEC Imaging Systems GmbH, Unterschleißheim, Germany), a commercially available, vendor-neutral software platform for 3DE quantification, performed by a single expert for the 27 test subjects (including rescans).

### 2.6. Statistics

Paired-sample *t*-tests were used to identify statistically significant measurement biases (calculated as index_3DE_–index_CMR_) in cardiac indices derived from 3DE (either by *nnU-Net* or manually) with respect to those obtained from corresponding CMR analyses, and Bland-Altman plots were used to visualize the agreement between paired variables. The *f*-test of equality of variances was used to assess the significance of the reduction in the standard deviation of errors when using *nnU-Net* instead of expert manual analyses in terms of measurement accuracy (with respect to CMR), as well as scan-rescan repeatability. Finally, to assess the reliability between paired measurements, an intraclass correlation coefficient (ICC) using a two-way, mixed effects model for absolute agreement, was calculated for each index. Based on established guidelines (33), threshold values of <0.5, ≥0.5, ≥0.75, and ≥0.9, represented poor, moderate, good, and excellent reliability, respectively. For the quantification of absolute scan-rescan variability due to random measurement error (34), repeatability coefficients with 95% confidence (35) were also computed. All statistical tests were two-tailed and deemed significant for *p*-values < 0.05, and analyses were performed using IBM SPSS Statistics for Windows (Version 26.0, IBM Corp., Armonk, NY, USA).

## 3. Results

### 3.1. Population summary

Demographics (including age, sex, and body surface area) and CMR-derived LV indices for the included population are summarized in Table 1. The disease group comprised 14 patients with LV hypertrophy, 12 patients with cardiac amyloidosis, 10 patients with aortic regurgitation, eight patients with hypertrophic cardiomyopathy, six patients with dilated cardiomyopathy, and two heart transplant recipients.

**TABLE 1:** Summary of participant demographics including age, sex, body surface area (BSA) calculated using the Mosteller formula **(36)**, and body mass index (BMI); and indices derived from cardiac magnetic resonance imaging including left ventricular end-diastolic volume (EDV), end-systolic volume (ESV), mass (LVM), and ejection fraction (EF), for the included dataset.

	Control (*n* = 82)	Disease (*n* = 52)	Total (*n* = 134)
Age (years)	37 ± 16 (18–74)	62 ± 15 (18–84)	47 ± 20 (18–84)
Male sex [frequency (%)]	42 (51%)	39 (75%)	81 (60%)
BSA (m^2^)	1.83 ± 0.21 (1.39–2.25)	2.01 ± 0.25 (1.46–2.72)	1.90 ± 0.24 (1.39–2.72)
BMI (kg/m^2^)	24.0 ± 3.6 (16.9–34.2)	28.3 ± 5.5 (16.7–48.9)	25.7 ± 4.9 (16.7–48.9)
EDV (ml)	139 ± 31 (74–220)	166 ± 44 (101–314)	150 ± 39 (74–314)
ESV (ml)	53 ± 16 (19–103)	74 ± 38 (29–235)	61 ± 29 (19–235)
LVM (g)	110 ± 30 (58–171)	170 ± 51 (88–314)	133 ± 49 (58–314)
EF (%)	62 ± 5 (51–74)	57 ± 12 (25–78)	60 ± 9 (25–78)
HR difference (bpm)	−1 ± 7 (−22–25)	−1 ± 6 (−13–38)	−1 ± 6 (−22–38)

The difference in heart rate (HR) between 3DE and CMR acquisitions (calculated as HR_3DE_–HR_CMR_) is provided as an indication of HR variability between modalities. Continuous variables are presented as mean ± standard deviation (range).

### 3.2. Image characteristics

**FIGURE 6:**
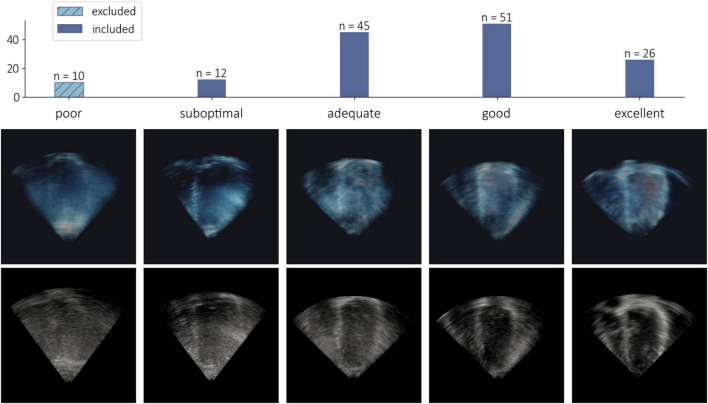
Examples of reconstructed 3D echocardiographic image volumes (visualized with an opacity transfer function on a blue-to-red colormap) and corresponding 2D mid-volume longitudinal slices (grayscale), showing variable quality (subjectively scored from poor to excellent). A total of 10 subjects were excluded from the study due to poor image quality.

Images of at least suboptimal quality (*n* = 134) were included for analysis, leaving 10 datasets that were excluded due to poor quality. Figure 6 shows examples of 3DE images ranging from poor to excellent quality, as well as the distribution of image quality across the population. Of the 10 excluded cases, five were healthy controls, and five were patients with cardiac disease. A summary of 3DE image dimensions and acquired frames per cycle is presented in Table 2.

**TABLE 2:** Summary of 3D echocardiography (**3DE)** image parameters including Cartesian image dimensions in *X* (elevation), *Y* (azimuth), *Z* (depth, i.e., apex-to-base) directions, the number of frames acquired per cycle, and the contrast-to-noise-ratio (CNR) associated with subjective quality scores across the included study population.

	Dimensions (mm)			Frames per cycle	CNR (dB)			
*n* = 268	*X*	*Y*	*Z*		Suboptimal	Adequate	Good	Excellent
Mean	167	168	132	36	0.526	0.649	0.831	0.930
SD	25	26	14	12	0.110	0.144	0.148	0.146
Min.	106	117	101	12	0.346	0.209	0.480	0.675
Max.	243	243	172	69	0.726	0.920	1.128	1.377

Presented values include the mean, standard deviation (SD), minimum (min.), and maximum (max.) for each parameter.

### 3.3. Segmentation accuracy

**FIGURE 7:**
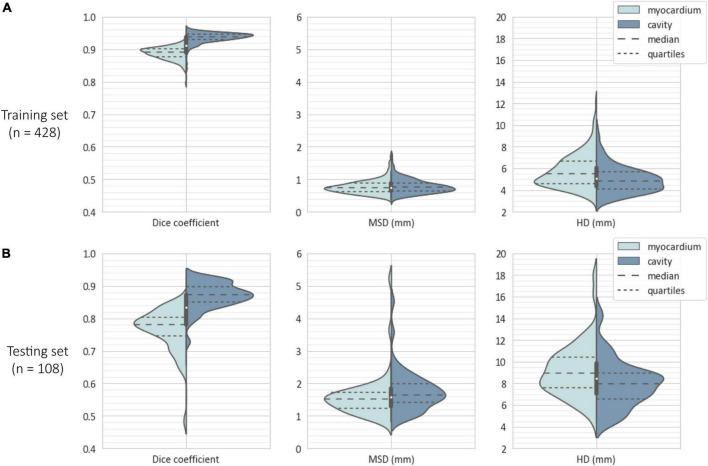
Violin plots showing the distribution and quartiles of segmentation scores in terms of Dice coefficient, mean surface distance (MSD), and Hausdorff distance (HD), evaluated on **(A)** the training set (*n* = 428 images) and **(B)** the testing set (*n* = 108 images). For each metric, distributions are split into the two foreground classes (i.e., myocardium and cavity), with the central box plot derived from the data of both classes as an estimate of the overall score.

Figure 7 illustrates the distribution of segmentation accuracy scores obtained by the ensembled *nnU-Net* model with respect to the cavity and myocardium, evaluated on the training set (*n* = 428, consisting of data from 107 subjects × 2 clips × 2 frames) and testing set (*n* = 108 images, consisting of data from 27 subjects × 2 clips × 2 frames). Mean test scores were Dice coefficient = 0.766, MSD = 1.6 mm, and HD = 9.1 mm for the myocardium; and Dice coefficient = 0.871, MSD = 1.8 mm, and HD = 8.0 mm, for the cavity. Segmentation metrics for each of the five separate model instances evaluated on the testing set is provided in the Supplementary material. For comparison, corresponding mean scores (averaged between the reported values for ED and ES) obtained by the most accurate method for LV cavity segmentation in the fully automatic category by Barbosa et al. (37) and Queirós et al. (38) of the 2014 MICCAI CETUS challenge were Dice coefficient = 0.878, MSD = 2.4 mm, and HD = 8.2 mm.

**FIGURE 8:**
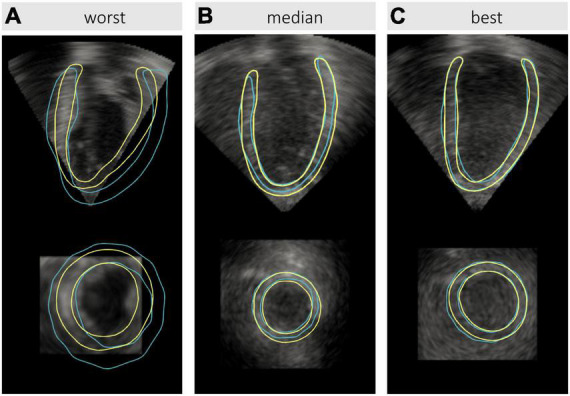
Comparison of left ventricular (LV) segmentations by *nnU-Net* (yellow) against reference labels (blue) derived from cardiac magnetic resonance (CMR) imaging for test images at end-diastole, representing the: **(A)** worst, **(B)** median, and **(C)** best model performances. For visualization purposes, 3D masks have been converted to contours representing the LV myocardium corresponding to longitudinal (top row) and axial (bottom row) slices. The resulting Dice coefficients for the myocardium and cavity were: 0.440 and 0.754, respectively, for the worst case; 0.741 and 0.924, respectively, for the median case; and 0.856 and 0.950, respectively, for the best case.

From visual assessment, *nnU-Net* produced reasonable myocardium and cavity segmentations for all test images at both ED and ES. Mis-segmentations occurred most frequently where LV boundaries were missing from the image, with one such example illustrated in Figure 8A. Here, the reference annotations show that a substantial portion of the cavity and myocardium falls outside the acquired pyramidal volume. Where the pyramidal volume adequately encompassed the LV, segmentations were generally accurate, as shown in Figures 8B, C.

### 3.4. Agreement in cardiac indices

**FIGURE 9:**
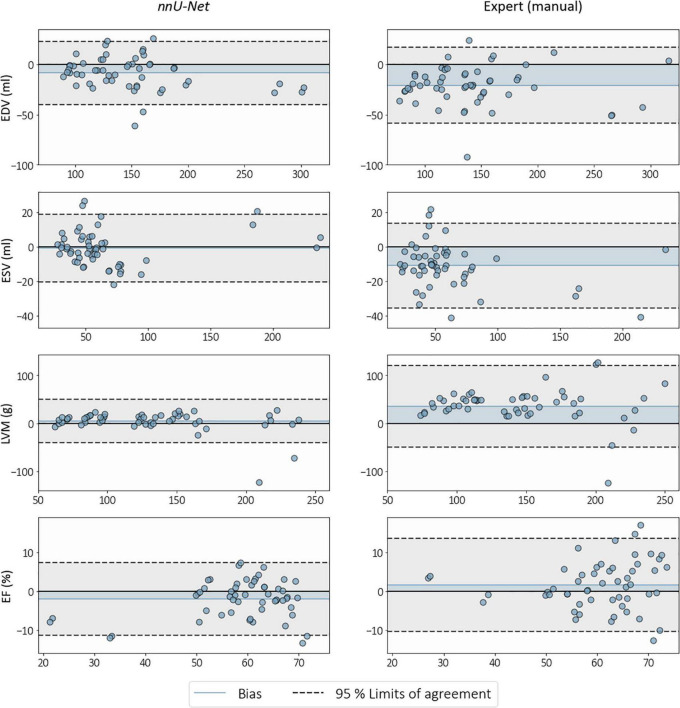
Bland-Altman plots showing biases and 95% limits of agreement between cardiac magnetic resonance (CMR) and 3D echocardiography (3DE) when analyzed by an expert and with *nnU-Net*. The horizontal axis represents the mean of measurements obtained from 3DE and CMR, against differences (calculated as 3DE–CMR) on the vertical axis, for end-diastolic volume (EDV), end-systolic volume (ESV), left ventricular mass (LVM), and ejection fraction (EF). Blue shaded regions represent the magnitude of bias from zero.

Agreement and reliability in clinical cardiac indices between CMR and 3DE (calculated from *nnU-Net* segmentations and expert manual analyses using TOMTEC) are presented in Table 3. Higher ICC values (representing measurement reliability with respect to CMR) were observed across all cardiac indices, with significant reductions in the magnitude of bias for EDV, ESV, and LVM when using *nnU-Net* in place of expert manual analyses for recovering CMR-derived cardiac indices. Bland-Altman analyses revealed narrower 95% limits of agreement in all cardiac indices for *nnU-Net* compared to expert manual analyses, with no apparent proportional bias (Figure 9).

**TABLE 3:** Left ventricular end-diastolic volume (EDV), end-systolic volume (ESV), mass (LVM), and ejection fraction (EF) for the testing set (*n* = 54 clips) derived from cardiac magnetic resonance (CMR), corresponding 3D echocardiography (3DE) measurement biases [mean ± standard deviation (SD)], and single measures intraclass correlation coefficients (ICC) with 95% confidence intervals in squared brackets.

	CMR	*nnU-Net*		Expert (manual)		Comparison	
*N* = 54	Mean ± SD	Bias	ICC	Bias	ICC	*t*-test	*f*-test
EDV (ml)	153 ± 52	*−9 ± 16	0.936 [0.855, 0.968]	*−21 ± 19	0.864 [0.301, 0.953]	**<0.001**	0.189
ESV (ml)	66 ± 44	−1 ± 10	0.975 [0.957, 0.985]	*−11 ± 13	0.927 [0.680, 0.972]	**<0.001**	0.104
LVM (g)	127 ± 55	5 ± 23	0.897 [0.830, 0.939]	*35 ± 43	0.532 [0.110, 0.754]	**<0.001**	**<0.001**
EF (%)	60 ± 10	*−2 ± 5	0.889 [0.795, 0.938]	2 ± 6	0.825 [0.714, 0.895]	**<0.001**	0.069

Values in bold in the Comparison column represent statistically significant differences (*p* < 0.05) between the means (*t*-test) and variances (*f*-test) of measurement biases for the expert manual and *nnU-Net* analyses. Asterisks (*) indicate statistically significant differences between 3DE and CMR using a paired *t*-test.

### 3.5. Scan-rescan repeatability

The variability in repeated 3DE measurements is summarized in Table 4. Both expert manual analyses and *nnU-Net* exhibited excellent reliability between scan-rescan measurements (ICC > 0.9), with the reliability of *nnU-Net* being higher for all cardiac indices. Similarly, *nnU-Net* outperformed the expert human observer in terms of significantly smaller magnitudes of variance in scan-rescan biases (again for all cardiac indices), suggesting that measurements obtained using *nnU-Net* were more consistent.

**TABLE 4:** Scan-rescan variability in left ventricular end-diastolic volume (EDV), end-systolic volume (ESV), mass (LVM), and ejection fraction (EF) for the testing set (*n* = 27 patients) in terms of measurement biases (calculated as randomized first measurement–second measurement), average measures intraclass correlation coefficients (ICC) with 95% confidence intervals in squared brackets, and 95% confidence repeatability coefficients (RC), derived from expert manual analyses and *nnU-Net* segmentations.

	*nnU-Net*			Expert (manual)			Comparison
*n* = 27	Bias	ICC	RC	Bias	ICC	RC	*f*-test
EDV (ml)	1 ± 9	0.991 [0.980, 0.996]	±18	2 ± 17	0.968 [0.930, 0.985]	±34	**0.002**
ESV (ml)	−1 ± 5	0.997 [0.994, 0.999]	±9	1 ± 10	0.982 [0.962, 0.992]	±20	**<0.001**
LVM (g)	1 ± 12	0.984 [0.965, 0.993]	±24	1 ± 24	0.944 [0.877, 0.975]	±47	**0.001**
EF (%)	1 ± 3	0.987 [0.971, 0.994]	±5	−1 ± 4	0.957 [0.907, 0.980]	±9	**0.005**

Values in bold in the Comparison column represent statistically significant differences (*p* < 0.05) between the variances of the biases.

## 4. Discussion

Guidelines and recommendations for LV chamber quantification using echocardiography state 3DE as the preferred method of volumetric assessment (over conventional 2DE), where available and feasible (39), in keeping with the advantage of 3DE in being able to circumvent the need for geometric assumptions. Recently published normative values stratified by age, sex, and ethnic groups by the World Alliance Societies of Echocardiography (WASE) (40) further endorses the use of 3DE for the assessment of LV chamber size and function. Nevertheless, 3DE has not yet been universally incorporated into standard clinical routine due to requiring specialized expertise in both acquisition and analysis, resulting in higher costs compared to 2DE. Likewise, the generation of large amounts of expert manual annotations for the development of automated 3DE analysis methods has historically been a tedious and complex task. Having recognized the inter-expert variability in manual analysis (such as was experienced during the organization of the CETUS challenge), we sought to instead leverage the higher resolution and contrast of CMR in a supervised manner, to provide more objective reference labels for 3DE. Furthermore, the use of CMR-derived labels provides an implicit advantage over manual 3DE segmentations in terms of reducing intermodality measurement bias.

Using 536 annotated 3DE images from a heterogeneous population of 134 human subjects comprising healthy controls and patients with cardiac disease, the dataset was used to train a self-configuring 3D U-Net to provide automated segmentations of the LV cavity and myocardium at ED and ES. This automated *nnU-Net* model subsequently outperformed an expert human observer in terms of accuracy against CMR reference values, as well as scan-rescan repeatability, whilst exhibiting increased measurement reliability (in terms of ICC) for all measured indices. Compared to volumes obtained using conventional manual analyses, *nnU-Net* had a lower magnitude of bias between 3DE and CMR, by 12 ml for EDV, and 10 ml for ESV. Most markedly, myocardial mass estimates using *nnU-Net* were far superior to those obtained by manual analyses. The automated method produced a bias that was seven times smaller in magnitude (5 g *nnU-Net* bias compared to 35 g manual bias in Table 3) for LVM, and excellent reliability with respect to CMR (where previously only moderate reliability was attained using the manual method). While there were indeed statistically significant differences in mean EDV and EF values between *nnU-Net* and CMR, these differences (i.e., 9 ml and 2%, for EDV and EF, respectively) are clinically acceptable (41), and unlikely to influence diagnostic outcomes or treatment pathways. In terms of segmentation accuracy, *nnU-Net* achieved a comparable Dice coefficient for the LV cavity with lower MSD and HD scores compared to the highest-ranking method trained and evaluated on the CETUS dataset. However, it should be noted that these comparisons are indicative only, as results were obtained from evaluation on a different dataset.

Signal dropout [particularly at the anterior wall (10)] remains a major challenge in 3DE analysis. Furthermore, highly anisotropic speckle properties and decreasing lateral resolution (being inversely proportional to transducer proximity) obscures the boundary between the myocardium and cavity toward the base of the LV when imaged from the apical window. By leveraging subject-specific geometries from CMR, our approach provides reliable reference annotations in such regions that are otherwise unavailable. Compared to the use of population priors, subject-specific information is more likely to produce labels closer to the true LV geometry for a given image instance, which may be leveraged by computational classifiers such as convolutional neural networks, despite not being apparent to human observers. Although this is possible in the presence of low contrast or poor resolution, it remains a challenge for the ML model to predict labels in regions where image data is entirely absent, such as that illustrated in Figure 8A. This highlights the importance of image quality in terms of both texture as well as the selection of an appropriate pyramidal volume width during acquisition, the latter of which may result in a total lack of image information, and subsequent inability to recover geometric information.

The use of CMR-derived labels for 3DE relies on the assumption that there is no change in LV geometry (and associated hemodynamic status) between modalities. Although paired datasets were acquired with minimal time between CMR and 3DE scans, multimodal imaging was nevertheless performed asynchronously, with participants subject to natural physiological (e.g., heart rate) and positional (i.e., supine during CMR and lateral during 3DE) variability. Furthermore, different lung volumes during the breath-hold requirements for imaging may also influence venous return and consequently cardiac output (42). Thus, the assumption that LV volumes are identical for the same subject between scans consequently remains a limitation of the described method for the utilization of labels from a different modality. As the registration between CMR and 3DE only accounts for the rigid transformation component between imaging coordinate systems, it may be appropriate to incorporate affine components (such as scaling) to account for changes in LV geometry as a result of acquisition conditions. However, such changes are typically subtle for subjects at rest (43–45).

From a practical perspective, there are several advantages of using ML for 3DE analysis, including the reduction in the time required for analysis (with network inference time being approximately six seconds per 3DE image) and scan-rescan variability when compared with conventional methods, as exemplified in this study. The use of CMR in the creation of training data for automated 3DE analysis methods not only removes the measurement bias between the two modalities, but also provides more accurate and reproducible measurements (compared to manual analysis methods) to facilitate integration of 3DE into clinical practice. Lastly, the methodology surrounding the derivation of subject-specific labels from an alternative imaging modality is not limited to the LV, and similar approaches may be taken for other cardiac structures, such as the RV and cardiac atria, to enable more comprehensive examinations using 3DE.

### 4.1. Limitations and future work

While this work represents the largest publicly available 3DE dataset in terms of the number of labeled images, it currently stands as a single-center, single-vendor study (unlike CETUS, which includes data from three institutions and three ultrasound vendors). Similarly, reference geometries were obtained by a single observer, who performed both the CMR analysis [although interobserver variability is generally low (46)] as well as the manual refinement of CMR-to-3DE alignment. The reliance on a single observer consequently remains a limitation of this study, and further validation using an independent dataset is needed to assess the reproducibility of the label generation framework and overall robustness of the proposed method. Contributions from other institutions may also help to provide additional data variability to improve the generalizability and performance of the ML workflow presented here.

Although the use of 3D Cartesian images with isotropic spacing provides a standard format for input into most ML architectures, it is worth noting that in the case of 3DE, approximately two-thirds of the image consists of zero-values outside the pyramidal volume as a result of the rectangular bounding box. This redundancy warrants investigation into more efficient image representations and potential analysis on un-interpolated radiofrequency data, which may improve model performance.

As the present dataset is inclusive of ED and ES images only, this method may be extended to include intermediary frames and leverage temporal information (47) to enable automated full-cycle analysis. Such data would enable more in-depth analysis of cardiac motion or the assessment of diastolic function for added clinical value.

## 5. Conclusion

In light of the ongoing efforts in developing and evaluating automated 3DE analysis methods, we present here an annotated 3DE dataset comprising images of varying quality acquired across a range of patient demographics, representing the largest publicly available 3DE dataset to date, and the first of which leverages subject specific labels from CMR. Using this dataset, a state-of-the-art deep learning model applied to unseen 3DE images was capable of reproducing measurements derived from CMR, while outperforming an expert human observer in terms of accuracy and scan-rescan repeatability. As 3DE becomes increasingly widespread, the provision of a novel benchmark represents a critical step toward enabling the development of automated tools for enhanced efficiency and accuracy of non-invasive cardiac image analysis.

## Data availability statement

The datasets presented in this study can be found in online repositories. The names of the repository/repositories and accession number(s) can be found below: https://www.cardiacatlas.org/.

## Ethics statement

The studies involving human participants were reviewed and approved by the Health and Disability Ethics Committee (HDEC) of New Zealand (17/CEN/226). The patients/participants provided their written informed consent to participate in this study.

## Author contributions

DZ devised the data generation framework, performed manual analyses and registrations, carried out the deep learning experiment, and drafted the initial manuscript. EF and GM advised on the deep learning aspects. GQ acquired the 3DE data and provided technical imaging expertise and manual segmentations. KG and VW established the pilot data acquisition protocol. TB advised on data sharing and management. JP and JD’h developed the BEAS algorithm. TS, BL, ML, PR, and RD contributed to patient recruitment and provided clinical expertise. OC advised on the interpretation and presentation of results. AY and MN designed the study, co-supervised the research, and assisted with interpretation of results. All authors have contributed significantly to the submitted work, including involvement in the research design, analysis and interpretation of data, and critical revision of the manuscript draft.

## Footnotes

1
https://www.creatis.insa-lyon.fr/Challenge/CETUS/

2
https://www.cardiacatlas.org/
