# Supplementary material for: MITEA: A dataset for machine learning segmentation of the left ventricle in 3D echocardiography using subject-specific labels from cardiac magnetic resonance imaging
Source: Front Cardiovasc Med. 2023 Jan 10;9:1016703. doi: 10.3389/fcvm.2022.1016703 (PMC9871929; doi:10.3389/fcvm.2022.1016703)
Supplement: Supplementary file 1 [file Table_1.DOCX]

Supplementary Material

# Supplementary Data

Supplementary Table I. Segmentation scores produced by each of the separate model instances trained during five-fold cross-validation, as well as the final ensembled *nnU-Net* model, in terms of Dice coefficient, mean surface distance (MSD), and Hausdorff distance (HD). Evaluation is performed using the same testing set (N = 108 images), and values are presented as mean ± standard deviation.

| **Model** | **Myocardium** | | | **Cavity** | | |
| --- | --- | --- | --- | --- | --- | --- |
| **N = 108** | **Dice** | **MSD** | **HD** | **Dice** | **MSD** | **HD** |
| Fold #1 | 0.761 ± 0.061 | 1.6 ± 0.6 | 9.4 ± 2.3 | 0.869 ± 0.039 | 1.8 ± 0.6 | 8.1 ± 2.2 |
| Fold #2 | 0.753 ± 0.077 | 1.7 ± 0.8 | 9.5 ± 3.0 | 0.863 ± 0.045 | 1.9 ± 0.8 | 8.3 ± 2.5 |
| Fold #3 | 0.757 ± 0.071 | 1.6 ± 0.7 | 9.5 ± 2.6 | 0.866 ± 0.041 | 1.8 ± 0.7 | 8.3 ± 2.3 |
| Fold #4 | 0.762 ± 0.063 | 1.6 ± 0.6 | 9.5 ± 2.6 | 0.867 ± 0.041 | 1.8 ± 0.6 | 8.2 ± 2.4 |
| Fold #5 | 0.763 ± 0.057 | 1.6 ± 0.6 | 9.4 ± 2.5 | 0.869 ± 0.037 | 1.8 ± 0.6 | 8.1 ± 2.2 |
| **Ensemble** | 0.766 ± 0.064 | 1.6 ± 0.7 | 9.1 ± 2.5 | 0.871 ± 0.040 | 1.8 ± 0.6 | 8.0 ± 2.2 |
